# Learning from *Ananya*: Lessons for primary health care performance improvement

**DOI:** 10.7189/jogh.10.020356

**Published:** 2020-12

**Authors:** Gary L Darmstadt, on behalf of the Ananya Study Group

**Affiliations:** Department of Pediatrics, Stanford University School of Medicine, Stanford, California, USA

## CONTEXT

The *Ananya* program is a highly ambitious and courageous effort to improve reproductive, maternal, newborn and child health and nutrition (RMNCHN) statewide in Bihar, India, across a population of approximately 120 million people [1], in an extremely complex, challenging environment [2]. In 2010 when *Ananya* was initiated, Bihar was emerging from decades of political upheaval and weak governance and leadership; faced crippling shortfalls of health infrastructure, commodities, management and staffing, and was among the lowest performing of Indian states for RMNCHN indicators. A*nanya* was launched by the Government of Bihar (GoB) and the Bill and Melinda Gates Foundation (BMGF) with strong political will and commitment to health sector performance improvement. The GoB and BMGF set ambitious goals focused on reducing maternal, neonatal, infant and under-five child mortality and childhood malnutrition. BMGF made grants to multiple organisations for supply and demand-side interventions and attempted to unify these activities under a common theory of change [2]. These activities were facilitated through regular partner convenings including the GoB.

## IMPLEMENTATION

The first phase of the program included intensive ancillary support by non-governmental organisations (NGOs) to governmental implementation in a sizeable population of about 28 million in eight focus districts [2]. BMGF gave its grantees the rare opportunity of substantial leeway to develop and test innovations, both technical and managerial [3-5]. After a two-year pilot phase, the GoB launched the scale-up of selected interventions statewide, which necessitated that non-governmental partners transition to a new structure for provision of technical and managerial support through the Bihar Technical Support Program and scale-back on-the-ground presence to about 25% of phase 1 capacity.

## CAPTURE OF LEARNING

Stanford University led an effort with the *Ananya* partners, bringing together policy, programmatic and research expertise – including the BMGF India Country Office, CARE India, BBC Media Action, Project Concern International, Population Council, Mathematica, Johns Hopkins University, the University of California San Francisco, and George Washington University – to capture learning from the two-year pilot (2012-2013) and the first four-years of statewide scale-up (2014-2017) of *Ananya* technical and managerial innovations, interventions and delivery approaches across the RMNCHN continuum of care. Sixteen manuscripts in addition to this commentary have resulted from this comprehensive analysis, twelve of which appear as a special collection in the *Journal of Global Health*: “*Ananya* : Lessons from Primary Health Care Performance Improvement.”

## KEY FINDINGS

### Pilot phase and experimental trials

A focus of the pilot phase was on experimentation with innovations to vexing challenges in advancing RMNCHN at scale. During this period, CARE India designed and rigorously tested team-based incentives, non-monetary incentives and promotion of team-based performance improvement which showed promise as an approach for improving frontline worker (FLW) coordination and teamwork and the quality and quantity of FLW-beneficiary interactions [2]. For instance, FLW use of a customised mHealth tool was associated with more frequent antenatal and postnatal home visits and greater job confidence among FLWs, as well as improved RMNCHN behaviours among beneficiaries [4].

The pilot phase was also an intensive period of intervention development, piloting and testing by all *Ananya* partners. A quasi-experimental independent evaluation by Mathematica of overall impacts of the first phase of *Ananya* revealed that secular changes were under way in nearly half of RMNCHN indicators, unrelated to *Ananya* [6]. Additional changes attributable to the *Ananya* program were found for about 10% of indicators and were most prominent for mothers’ decisions around contraceptive utilisation. However, the evaluation was not powered to observe statistically significant differences in RMNCHN indicators within two years, and could not measure changes due to specific interventions, but rather was aimed to help inform program improvements and scale-up.

A key learning of the eight-district pilot phase was that deep structural constraints in health system organisation and delivery of interventions posed substantial limitations on behaviour change among health care providers and beneficiaries. Qualitative research suggested that while Accredited Social Health Activists (community-based female health workers) are deeply engaged in community service, they face pervasive delays in incentive payments, increased workload, lack of training and integration with the rest of the health system, and punishing managerial and supervisory practices which fuel job dissatisfaction [7]. CARE India shifted focus after the pilot phase of *Ananya* from building capacity of the FLW platform to developing innovations and addressing fundamentals of health system strengthening and quality improvement in health facilities.

### Statewide scale-up

These early results provided a signal that large-scale change was possible under conditions of intensive support to implementation, bolstered by innovative solutions identified through rigorous, expansive experimentation. The GoB forged ahead with scale-up efforts across the rest of the thirty districts in the state, assuming full ownership for program implementation. Going forward, however, it became impossible to maintain a control group or a quasi-experimental evaluation. Thus, evaluation of impact became dependent primarily on a series of Community-based Household Surveys conducted by CARE India primarily for the purpose of program monitoring rather than impact evaluation. Without a control group, full attribution of change to *Ananya* was no longer possible.

When ancillary, on-the-ground experimentation and presence of *Ananya* partners was pulled back during statewide scale-up and they focused on providing techno-managerial support to the government, gains in RMNCHN that had been achieved in the pilot phase rapidly fell back to baseline for the majority of RMNCHN indicators [8]. In general, over the course of the next four years (2014-2017) during the scale-up phase, indicators in the eight original focus districts did not dip below baseline levels and the response to implementation at scale under the BTSP was similar in the former focus and non-focus districts. This suggests that health systems had been neither strengthened nor further destabilised in the first phase of *Ananya*.

As implementation was scaled up statewide by the GoB, changes in RMNCHN indicators measured statewide were mixed with increases in some indicators balanced by decreases in others [8]. Most notably, analysis of changes in indicators at the block level revealed that average trends at the district-level masked considerable differences in block-level trends, even within the same district [9]. This highlights the importance of understanding local factors driving or impeding change, signals caution in making implementation decisions based only on the average statewide or even district-level trends, and emphasises the need for greater precision in targeting interventions based on real-time measures over space and time to identify where to intensify delivery of which interventions to address local contextual and/or programmatic barriers. Of note, facility delivery, especially within public facilities, was exceptional in showing little variation in trends over time, overall and geospatially. Thus, despite increased global, national and local emphasis on the importance of skilled care at delivery, home births remain important to address in Bihar.

During the scale-up phase, *Ananya* partners engaged in intensive efforts to inform approaches to achieve impact at scale through health systems strengthening. CARE India partnered with the GoB to develop and scale-up a wide array of managerial and technical solutions, including improvements in infrastructure, human resources management, supply chains, data systems and ultimately the quality of care in health facilities [10], with simulation and competency-based nurse mentoring in intrapartum and newborn care a cornerstone for progress [11,12]. Early results suggest that Bihar is poised for large-scale impact in RMNCHN through improved quality of care at facilities if scaled by government with sufficient intensity.

BBC Media Action experimented with the development and deployment of mHealth-based mass media and interpersonal communications in stimulating changes in RMNCHN knowledge and health-related behaviours. Overall reach of the Mobile Kunji tool, which facilitated knowledge transfer from FLWs to women, was low (8% of the population, 39% of beneficiaries reached by a FLW) [13]. Use of Mobile Kunji, however, was associated with increased FLW coordination and trust/credibility with beneficiaries and with improved RMNCHN knowledge and behaviours among those reached by FLWs with the tool. Rapid development, scale-up and handoff to the government of other mHealth tools in which alignment of user-driven design with government interests helped to drive scale-up – notably Kilkari (stage-specific audio content for mothers) and Mobile Academy (FLW training) in Bihar and elsewhere in India – evidences the exciting potential that exists for impact through application of mHealth [5,13].

**Figure:**
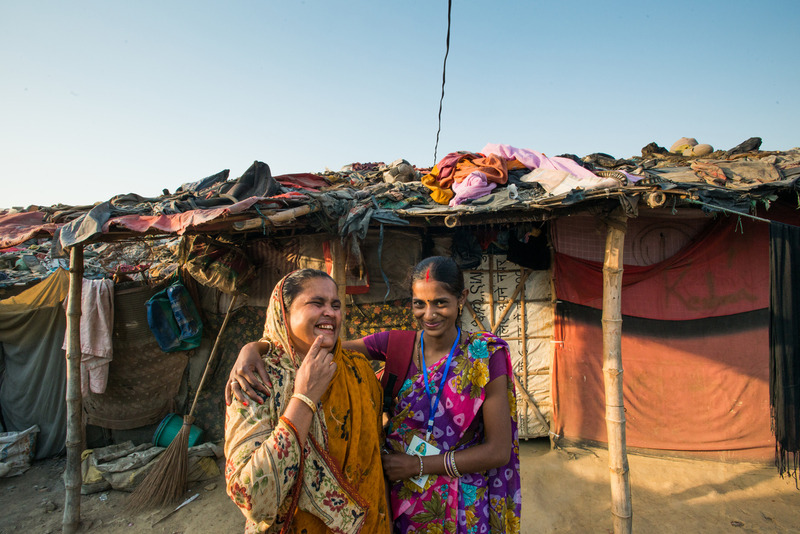
Photo: From the collection of the India Office, Bill and Melinda Gates Foundation (used with permission).

Innovations in self-help groups by Project Concern International to layer health interventions into groups originally conceived for promoting livelihoods and access to credit was shown in independent evaluations by Population Council and CARE India to be a scalable and highly effective model for broadly improving RMNCHN knowledge and behaviours [14,15]. The health layering model was subsequently adapted, integrated into, and shown to be associated with highly robust improvements in health across the RMNCHN continuum of care – especially antenatal care, birth preparedness, postnatal care and family planning – in government-led self-help groups across about one-quarter of the state [15]. This platform appears to be particularly promising for reaching marginalised households and communities.

These interventions may be especially important considering that our equity analysis revealed that changes in RMNCHN indicators were driven largely by changes in the least marginalised subgroup (by wealth and caste) of the population [16]. Essentially no change in indicators occurred among the most marginalised. Moreover, disparity tended to widen for indicators dependent on access to health facility care, suggesting that focus on facility-based care without also addressing barriers to access may improve indicators overall at the expense of accentuated inequality.

## REFLECTIONS

Investment in *Ananya* by BMGF, with high-level commitment by the GoB, created fertile ground for the development of numerous innovations in primary health care delivery at scale. Innovations included processes and tools for improving the effectiveness of FLWs in reaching and engaging with families in improving RMNCHN knowledge and behaviours, mHealth tools, health-layered SHGs, and facility quality improvement approaches such as nurse mentoring, team-building and use of simulations to improve intrapartum and newborn care. Insights into best practices for evaluation of complex global health programs in low- and middle-income countries emerged [17]. which may improve learning, policy and program impact.

The *Ananya* pilot demonstrated that with intensive ancillary support by NGOs to governmental implementation of primary health care interventions, substantial improvement in RMNCHN is possible. With continued governmental political will and commitment of resources, and with techno-managerial support from NGOs, GoB goals for health improvement are within reach.

In order to ensure these improvements reach across the population, there is a need to balance community and facility-based interventions. In the era of the Sustainable Development Goals, it is critical to take an equity lens in evaluations and to monitor for changes across socio-demographic groups to ensure that reach and impact are equitably distributed. Reach to the most marginalised with interventions targeted to address their needs must be intentional. Otherwise, an unintended consequence of universal primary health care programming, particularly if focused exclusively on facility-based processes and care, could be an accentuation of inequality, leaving the most marginalised further behind.

While clear health goals for *Ananya* were set, they were set at a higher level (ie, mortality reduction) than could be effectively tracked by the evaluations. The various partners had theories of change, but these were not necessarily aligned with each other or with a common set of measurable goals. Secular trends in health improvement in Bihar were strong, and without an ongoing ability to take this into account, including contextual factors and effects of other programs, attribution of change to *Ananya* program implementation was not possible, which may limit the strength of evidence for ongoing investment and policy change. Geospatial data on implementation progress was lacking and is important for associating implementation strength and the impacts of overlapping/intersecting interventions with changes in indicators over time. In the absence of a quasi-experimental evaluation design, the inability to calculate a dose response relating implementation to changes in indicators may further hinder the strength of evidence for influencing future policy change. It is also essential to measure change at the lowest possible implementation unit. Summary measures at district level, for example, may or may not provide insight into the contextual or programmatic factors associated with progress or lack thereof. Greater precision is needed in linking implementation factors and contextual factors to health measures, in order to gain insights into facilitating factors and barriers in improvement, or alternatively in decline, in health indicators. This would also enable analysis of outliers, seldom examined, but which may hold important insights into conditions which mediate change.

The GoB continues to partner with BMGF, CARE India, several of the *Ananya* NGOs, and others to advance RMNCHN and adolescent health at scale. *Ananya* revealed massive structural challenges in primary health care delivery in Bihar, but also demonstrated that with investments in technical and managerial innovations and use of data to guide performance improvement, accelerated change is possible if evidence-based programs are implemented with sufficient intensity. Going forward, it will be critical to balance investments across the facility-community continuum; to measure impacts at local (eg, block) level, disaggregated across intersecting aspects of marginalisation such as wealth, caste and gender; to maintain focus on the achievement of shared goals through a unifying, measurable theory of change; and to keep alive the *Ananya* spirit of optimism, urgency, innovation, and flexibility to evolve in response to ever-emerging quantitative and qualitative evidence of what is being achieved, for whom, where, and why or why not.
